# Interspecific variation in leaf functional and defensive traits in oak species and its underlying climatic drivers

**DOI:** 10.1371/journal.pone.0202548

**Published:** 2018-08-20

**Authors:** Luis Abdala-Roberts, Andrea Galmán, William K. Petry, Felisa Covelo, María de la Fuente, Gaétan Glauser, Xoaquín Moreira

**Affiliations:** 1 Departamento de Ecología Tropical, Campus de Ciencias Biológicas y Agropecuarias, Universidad Autónoma de Yucatán, Itzimná, Mérida, Yucatán, México; 2 Misión Biológica de Galicia (MBG-CSIC), Pontevedra, Galicia, Spain; 3 Institute of Integrative Biology, Eidgenössische Technische Hochschule (ETH) Zürich, Universitätstrasse 16, Zurich, Switzerland; 4 Departamento de Sistemas Físicos, Químicos y Naturales, Universidad Pablo de Olavide, Sevilla, Spain; 5 Neuchâtel Platform of Analytical Chemistry, University of Neuchâtel, Neuchâtel, Switzerland; Universidade Estadual Paulista Julio de Mesquita Filho, BRAZIL

## Abstract

Plants exhibit a diverse set of functional traits and ecological strategies which reflect an adaptation process to the biotic and abiotic components of the environment. The Plant Economic Spectrum organizes these traits along a continuum from conservative to acquisitive resource use strategies and shows how the abiotic environment governs a species’ position along the continuum. However, this framework does not typically account for leaf traits associated with herbivore resistance, despite fundamental metabolic links (and therefore co-variance) between resource use traits and defensive traits. Here we analyzed a suite of leaf traits associated with either resource use (specific leaf area [SLA], nutrients and water content) or defenses (phenolic compounds) for saplings of 11 species of oaks (*Quercus* spp.), and further investigated whether climatic variables underlie patterns of trait interspecific variation. An ordination of leaf traits revealed the primary axis of trait variation to be leaf economic spectrum traits associated with resource use (SLA, nitrogen, water content) in conjunction with a defensive trait (condensed tannins). Secondary and tertiary axes of trait variation were mainly associated with other defensive traits (lignins, flavonoids, and hydrolysable tannins). Within the primary axis we found a trade-off between resource use traits and both water content and condensed tannins; species with high SLA and leaf N values invested less in condensed tannins and viceversa. Moreover, temperature and precipitation mediated the trait space occupied by species, such that species distributed in warmer and drier climates had less leaf N, lower SLA, and more defenses (condensed tannins, lignins and flavonoids), whereas opposite values were observed for species distributed in colder and wetter climates. These results emphasize the role of abiotic controls over all-inclusive axes of trait variation and contribute to a more complete understanding of interspecific variation in plant functional strategies.

## Introduction

As plants have diversified, their adaptations to new environments have been recorded in their myriad growth forms and life histories [1]. However, these trait combinations found in nature comprise only a subset of the trait space. Comparisons of traits among plant species reveal that similar ecological strategies emerge in response to similar environmental pressures [2,3]. The Plant Economic Spectrum formalizes the expectations for trait correlations by showing how abiotic constraints yield a continuum of viable resource acquisition and allocation strategies ranging from “acquisitive” to “conservative” [4]. Acquisitive species are characterized by high growth rates and heavy allocation of resources to the construction of nutrient-rich tissues, whereas conservative species are slower-growing and invest in longer-lived tissues that offer lower, more sustained returns [1,5,6].

Plant tissues not only gather resources for the plant, but also serve as food resources to consumers. As such, plants also allocate considerable energy to the production of anti-herbivore defense compounds [7]. A number of theories have been proposed to explain interspecific variation in plant defense investment (reviewed by Stamp [8]), several of which invoke resource allocation trade-offs. Notably, the Resource Availability Hypothesis [9,10] poses that species in resource-poor environments invest more resources in defenses than in growth because replacement of plant tissues lost to herbivores is more costly when nutrients limit future growth and since the relative impact of herbivory increases with decreasing inherent growth rate. Viewed from the broader perspective of resource allocation in plants, it is clear that the biotic environment imposes additional constraints on plant traits that are unlikely to be independent of the abiotic constraints in the Plant Economic Spectrum because of the shared resource pools for metabolic and defensive functions [5, 6]. Despite this fundamental linkage between the construction of resource acquisition tissues and their defense, plant defenses are poorly integrated into the Plant Economic Spectrum (but see [6, 11]), limiting our understanding of how climate constrains plant defense and, in parallel, of how plant defense constrains broader suites of plant functional traits.

Environmental gradients represent useful proxies of multi-dimensional variation in biotic and abiotic factors [12,13]. Studies have often involved plant trait measurements along environmental gradients and an examination of underlying drivers of interspecific variation in both resource use (e.g. [5,14]) and defenses (e.g. [15,16]). For instance, climatic variables such as temperature and precipitation account for a large portion of interspecific variation in leaf nutrient content [14], and to some extent also functional traits [5] and secondary metabolites [15]. These studies therefore suggest an important component of abiotic control over plant ecological strategies, mediated directly or indirectly through effects on resource availability or enemy pressure. However, these evaluations of abiotic control over multi-dimensional plant trait variation have usually measured resource use or defense traits, but not both.

The present study was aimed at identifying all-inclusive axes of plant trait co-variation across 11 oak (*Quercus*) species. We chose this genus because its defensive and functional traits have been studied in the past for a number of species [17,18], and because species occupy contrasting habitats and exhibit different natural histories which can lead to interspecific variation in ecological strategies [19]. We conducted greenhouse measurements of a suite of eight leaf traits in one-year old saplings, four of which were associated to resource use (leaf water content, specific leaf area, nitrogen and phosphorus content) and the other four were related to chemical defense against herbivory (hydrolysable and condensed tannins, lignins, and flavonoids). The former group of traits measured have well-established relationships with the resource use (e.g. carbon uptake and investment), whereas phenolic compounds, despite representing an arguably narrow characterization of defenses, are the most important group of defensive secondary metabolites in *Quercus* and are therefore a good proxy of defense investment and costs [17]. In addition, we also extracted climatic data for each species based on their geographic distribution to assess the influence of abiotic components of the environment on patterns of plant trait variation. In doing this, we asked the following: (*i*) Are there detectable patterns of interspecific variation in leaf trait co-expression? In this case, we were interested in determining if traits or groups of traits traded off or not, and whether resource use and defensive traits separated into different axes (i.e. were decoupled) or not. And, (*ii*) Are any such patterns of trait variation underlain by abiotic correlates of climate (temperature and precipitation)? Whereas previous work has reported on patterns of interspecific variation in leaf defenses with respect to latitude and phylogenetic relatedness in the genus *Quercus* [18,19], here we aimed at explicitly integrating resource use and defensive traits to achieve a more complete understanding of all-inclusive functional strategies in oak species as well as other long-lived trees.

## Material and methods

### Ethics statement

The research did not involve manipulations of humans or animals. No specific permissions were required for our greenhouse work. The study did not involve endangered or protected species.

### Experimental conditions and measurements

We used 11 oak species for this study, namely: *Quercus robur* L., *Quercus faginea* Lam, *Quercus suber* L., *Quercus ilex* L., *Quercus pubescens* Willd., *Quercus pyrenaica* Willd., *Quercus agrifolia* Née, *Quercus macrocarpa* Michx., *Quercus rubra* L., *Quercus muehlenbergii* Engelm., and *Quercus palustris* Münchh. These species are distributed throughout Europe and North America (S1 Fig) and for each one we got seeds from a commercial supplier (Planfor nursery, Uchacq-et-Parentis, France). In August 2015 we sowed seeds in 4-L pots containing potting soil with peat in a glass greenhouse. A total of 12 plants per species (N = 132) were randomly allocated within each of 12 blocks. Plants were grown in a common environment under controlled light (minimum 12 h per day) and temperature (10°C night, 25°C day) with daily watering. In June 2016, 14 months after planting, all plants were harvested to quantify leaf traits.

### Quantification of leaf physical traits

Immediately after harvesting, we weighted all the fresh leaves of each plant and oven-dried the samples for 48 h at 40°C until constant weight was achieved. We then weighted the dry leaves and estimated leaf water content [(fresh weight-dry weight)/fresh weight] per plant. In addition, we calculated specific leaf area (SLA) for each plant by dividing the surface area of a 9.5-mm diameter disk by its dry mass in mg. We only measured a single leaf per plant because previous trials demonstrated relatively low among leaf variation within individual plants [20].

### Quantification of leaf chemical traits

After measuring physical traits, we ground the dried leaves of each plant in liquid nitrogen for quantification of nutrients and phenolic compounds. We chose phenolic compounds as defensive traits because they are widely demonstrated to be herbivore feeding deterrents across many plant taxa [15,21–23] and have been reported to confer resistance against insect herbivores in *Quercus* species specifically [24–27]. We extracted phenolic compounds using 20 mg of dry plant tissue with 1 mL of 70% methanol in an ultrasonic bath for 15 min, followed by centrifugation [15]. These methanolic extracts were then diluted (1:5 vol:vol) with the extraction solvent and transferred to chromatographic vials. We performed phenolic profiling according to Moreira et al. [28] with some modifications. Briefly, we used ultrahigh-pressure liquid chromatography-quadrupole-time-of-flight mass spectrometry (UHPLC-QTOF-MS) to detect, identify and quantify phenolic compounds. The separation was carried out on a 50 × 2.1 mm Acquity UPLC BEH C18 column (Waters, Milford, CT, USA) thermostated at 25°C. Solvents were A = water + 0.05% vol. formic acid; B = acetonitrile + 0.05% vol. formic acid. The gradient program was performed at a flow rate of 0.4 mL/min under the following conditions: 5–30% B in 6 min, 30–100% B in 2 min, holding at 100% B for 2 min followed by re-equilibration at 5% B for 2 min. The injection volume was 2 μl. The QTOF-MS was operated in MS^E^ negative mode over an m/z range of 85–1200 Da with the following parameters: capillary voltage at -2.5 kV, cone voltage -25 V, source temperature 120ºC, desolvation gas temperature 350ºC, desolvation gas flow 800 L/hr. Internal calibration of the instrument was obtained by infusing a solution of leucine-enkephaline at 400 ng/mL at a flow rate of 15 μL/min through a Lock Spray^TM^ probe. We identified phenolic compounds on the basis of their molecular formula (as determined from exact mass measurements), fragment ions, and comparison with available databases such as the Dictionary of Natural Products (Chapman & Hall, CRC Informa, London; version 20.2) and ReSpect for Phytochemicals [29]. We quantified flavonoids as rutin equivalents, condensed tannins as catechin equivalents, hydrolysable tannins as gallic acid equivalents, and lignins as ferulic acid equivalents. We achieved the quantification of these phenolic compounds by external calibration using calibration curves at 0.2, 0.8, 2, 5 and 20 μg/mL. We expressed phenolic compound concentrations in mg g^-1^ tissue on a dry weight basis.

For phosphorus and nitrogen, we digested approximately 0.1 g of ground dried leaf material in a mixture of selenous sulphuric acid and hydrogen peroxide [30]. Diluted aliquots of the digestion were analyzed by colorimetry for using the indophenol blue method in the case of nitrogen and the molybdenum blue method for phosphorus using a Biorad 650 microplate reader (Bio-Rad Laboratories, Philadelphia, PA, USA) at 650 nm and 700 nm, respectively [31]. We expressed nutrient concentrations as mg g^-1^ tissue on a dry weight basis.

### Species distribution range and climatic variables

For each of the 11 oak species, we constructed a species distribution models and extracted climatic data from the estimated species range. We obtained georeferenced species presence data from the Global Biodiversity Information Facility database (GBIF; https://www.gbif.org/). We cleaned the dataset by removing records that were incompletely georeferenced [32] occurred outside of the species native range as determined by the country names [European species] or state/province names [United States and Canada) listed in forest species atlases [33,34]. We used these occurrence data to construct a Maximum Entropy Species Distribution Model (SDM) for each oak species using the MaxEnt software [35] as implemented via the dismo package [36] for R v. 3.4.1 [37]. We divided the species presence data into five equal partitions and used k-fold cross-validation. Four of these partitions were used to train the models with 30-arcsecond resolution climate data from 19 BIOCLIM variables from the WorldClim database [38] as predictors. Pseudoabsences for the SDMs were generated within 10 km of occurrence points, determined by multiplying the maximum reported seed dispersal in oaks (4 km; [39]) by 2.5. This heuristic avoids the conflation of absences due to the environment itself with absences caused by dispersal limitation. The fifth data partition was used to assess the model fit using the area under the receiver-operator curve (AUC). For each oak species, this procedure was repeated such that each species presence data partition was used once as the validation dataset. We discretized the SDM by taking all areas with an occurrence probability ≥75% as the species range.

We quantified the average climate within each modelled species range. Specifically, we extracted the mean annual precipitation (mm) and mean annual temperature (°C) from the BIOCLIM data layers and used the median of each variable in our statistical analyses.

### Statistical analyses

First, we assessed the magnitude of species trait variation by fitting general linear mixed models (GLMMs) for each of the leaf traits with species as the independent variable (fixed effect) and block as a random effect. The purpose of these analyses was exclusively to quantify the univariate variation in each trait, as trait correlations prevent the meaningful application of inferential statistics. Second, we assessed multivariate patterns of interspecific variation in resource use and defensive traits using Principal Component Analysis (PCA). This ordination allowed us to derive independent axes of leaf trait variation and identify oak species falling on such axes, as well as determine whether traits structuring each axis traded off or were co-expressed [15,18]. All the above analyses were performed in SAS ver. 9.4 (SAS Institute, Cary NC). The GLMMs were run with the PROC GLM function and the PCA with the PROC FACTOR function (rotation = varimax). Residuals were normally distributed in all cases. Third, to test for the association between climate and the main axes of trait variation, we performed univariate regressions with temperature or precipitation as predictors of the standardized *z*-scores from the first three axes of the PCA for leaf traits. We subsequently controlled for phylogeny by performing phylogenetic correct generalized least square analyses (pGLS) with the pgls function in caper package in R [40] and present results from these analyses. These pGLS were based on a phylogenetic tree of *Quercus* species using Single Nucleotide Polymorphism matrices by ddRAD sequencing (S2 Fig).

## Results

### Species variation in leaf traits

In the case of traits associated with resource use, we found 1.6-fold (water content) to 11.2-fold (P concentration) variation among species, whereas in the case of defensive traits there was 4.9-fold (flavonoids) to 448-fold (condensed tannins) variation (Table 1).

**Table 1:** Oak species variation in leaf traits. Results from general linear mixed models to quantify variation among oak species in leaf traits associated with resource use (leaf water content, specific leaf area [SLA] in cm^2^ g^-1^, and nitrogen [N] and phosphorus [P] concentration in mg g^-1^ dry weight) or herbivore resistance (lignins, hydrolysable tannins, condensed tannins, and flavonoids, in mg g^-1^ dry weight). Descriptive statistics (least square means ± standard error and species range) are shown. Sample sizes per species = 12 in all cases (total N = 132). Inferential statistics are not appropriate due to strong multicollinearity between traits.

	Mean ± SE	Range
*Resource use traits*		
Water content	0.56 ± 0.006	0.37–0.72
SLA	0.36 ± 0.014	0.12–0.59
N	26.33 ± 0.66	14.92–34.89
P	2.33 ± 0.14	0.51–5.71
*Herbivore resistance traits*		
Condensed tannins	2.04 ± 0.33	0.02–10.57
Hydrolysable tannins	96.70 ± 7.89	4.52–196.36
Lignins	2.30 ± 0.18	0.37–4.82
Flavonoids	12.93 ± 0.76	4.34–21.18

### Multi-dimensional axes of leaf trait variation

**Fig 1:**
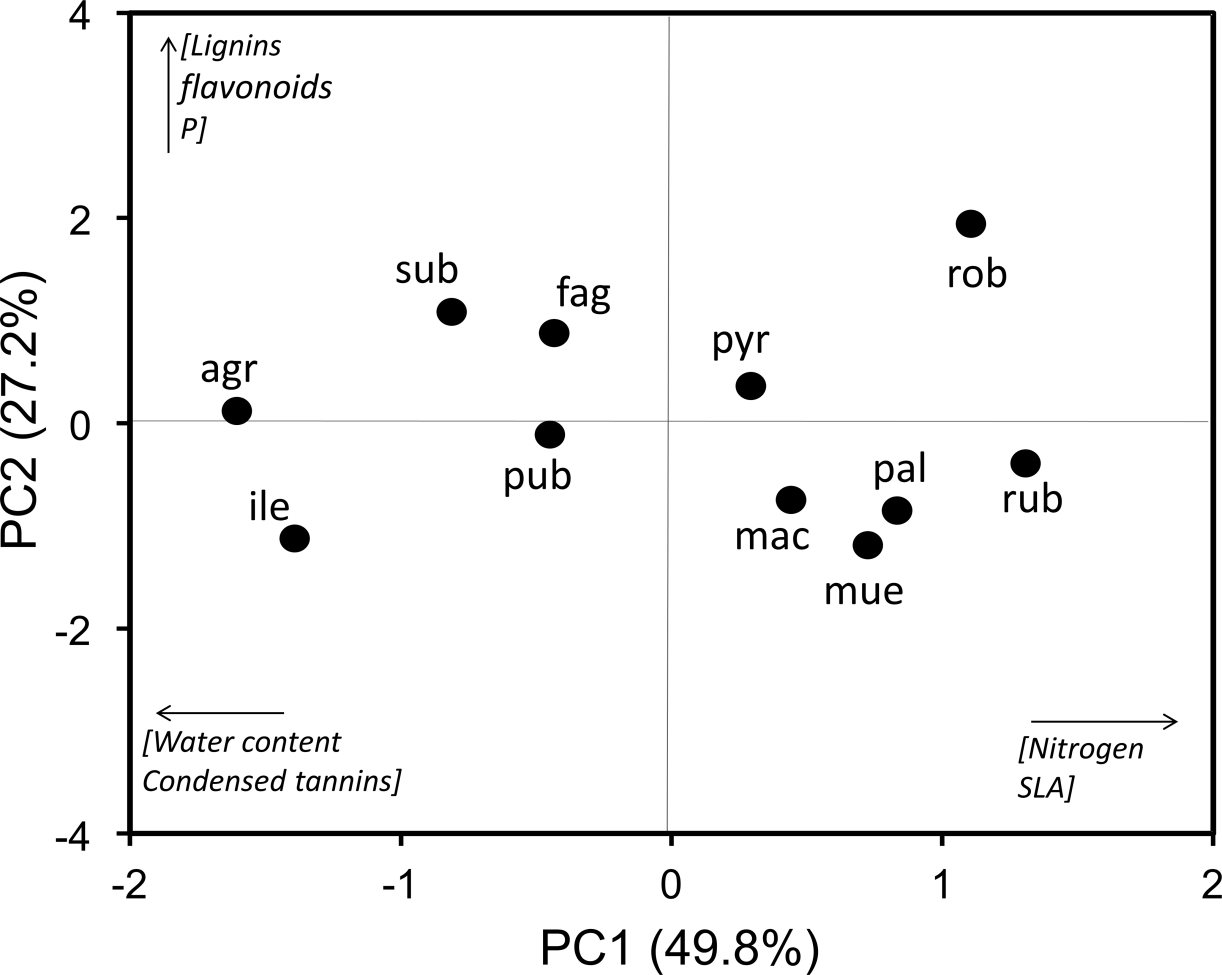
Principal components analysis of leaf traits. Principal components analysis for the 11 oak (*Quercus*) species based on a suite of traits associated with resource use or herbivore resistance. The most important traits associated with each axis are shown in brackets. Each circle represents species and text next to each circle is an abbreviation of the species names: agr = *Q*. *agrifolia*; fag = *Q*. *faginea*;; ile = *Q*. *ilex*; mac = *Q*. *macrocarpa*; mue = *Q*. *muehlenbergii*; pal = *Q*. *palustris*; pub = *Q*. *pubescens*; pyr = *Q*. *pyrenaica*; rob = *Q*. *robur*; rub = *Q*. *rubra*; sub = *Q*. *suber*.

Results from the ordination indicated that the first three axes explained 87% of the variation in the measured leaf traits (PC1 = 49.8%, PC2 = 27.2%, and PC3 = 10.1%). PC1 was strongly associated to water content, specific leaf area, nitrogen, and condensed tannins (Table 2; Fig 1). The sign of these loadings indicated that increasing values of PC1 were associated with decreasing leaf water content and condensed tannins on the one hand, and increasing SLA and N on the other (Table 2; Fig 1). PC2 was positively associated with leaf P, lignins and flavonoids, whereas PC3 was negatively associated with hydrolysable tannins (Table 2).

**Table 2:** Loadings of leaf traits on the first three axes of a principal components analysis. Loadings of oak leaf traits on the first three axes of a Principal Components Analysis including leaf traits associated with resource use (leaf water content, specific leaf area [SLA] in cm^2^ g^-1^, and nitrogen [N] and phosphorus [P] concentration in mg g^-1^ dry weight) or herbivore resistance (lignins, hydrolysable tannins, condensed tannins, and flavonoids, in mg g^-1^ dry weight). Traits with a strong loading (≥ 0.7) on a given PC are shown in bold.

	PC1	PC2	PC3
*Resource use traits*			
Water content	**-0.946**	-0.023	-0.273
SLA	**0.894**	-0.311	-0.152
N	**0.937**	0.043	0.239
P	0.510	**0.786**	-0.057
*Herbivore resistance traits*			
Condensed tannins	**-0.650**	-0.166	-0.599
Hydrolysable tannins	0.341	0.018	-**0.802**
Lignins	-0.404	**0.685**	0.441
Flavonoids	-0.139	**0.923**	0.025

### Effects of climate on main axes of trait variation

**Fig 2:**
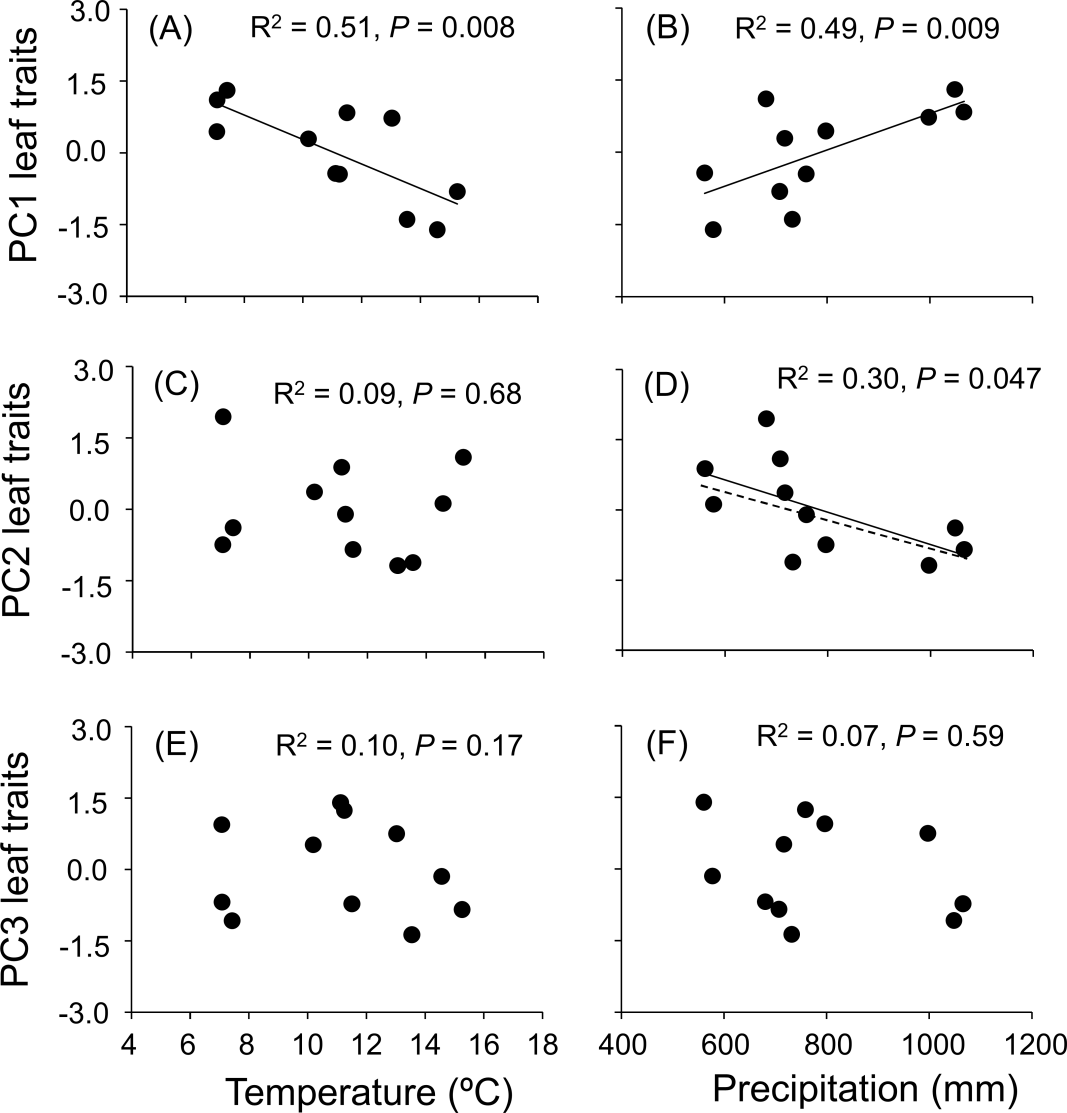
Relationships between climatic factors and leaf traits. Relationships between climatic mean annual temperature (°C) and mean annual precipitation (mm) (mean values based on a characterization of the climatic niche within the distribution range of each species; see *Methods*) with standardized *z*-score values from a Principal Components Analysis based on a suite of eight leaf traits associated with resource use or herbivore resistance (see *Methods*) measured across 11 oak species. Shown are the raw (solid) and phylogenetically independent (dashed) predicted relationships, and R^2^ and P-values are from to phylogenetically-corrected generalized least-square analyses.

Univariate regression analyses indicated that PC1 was significantly negatively associated with temperature and significantly positively associated with precipitation (Fig 2A and 2B). Based on the signs of the trait variables loading on PC1 (Table 2) these associations indicated that the leaves of oak species found in warmer climates had greater water content, lower SLA (thicker, tougher), lower N concentration, and a greater concentration of condensed tannins compared to the traits of species found in colder climates. Likewise, leaves from species distributed in wetter climates had lower water content and less condensed tannins but higher SLA (softer, thinner leaves) and greater nitrogen concentration. In addition, we found a significant negative effect of precipitation but no effect of temperature on PC2 (Fig 2C and 2D), indicating that species found in wetter climates had leaves with lower concentrations of P, lignin and flavonoids (traits structuring this axis). Finally, we found no association between temperature or precipitation and PC3 (Fig 2E and 2F), suggesting that interspecific variation in hydrolysable tannins (trait structuring this axis) is not influenced by climate.

## Discussion

We found high interspecific variation in leaf traits among the 11 oak species we studied, and moreover several suites of traits were strongly correlated. Three independent axes of variation explained > 80% of the co-variation in sapling leaf traits across the studied oak species. The first axis (PC1) was strongly associated with resource use traits (SLA, N, water content) but also with condensed tannins, a key group of secondary metabolites conferring herbivore resistance in *Quercus* [17,18,26,27]. Our finding that leaf resource use and defensive traits clustered together on the same axis of variation supports the few previous studies showing that both types of traits are frequently correlated and thus need to be jointly studied in order to better understand plant functional strategies [6,11]. A closer look at the traits associated with PC1 indicated that SLA and leaf N were positively associated with PC1 whereas water content and condensed tannins were negatively associated. The positive association between SLA and N is consistent with the predictions of the Plant Economic Spectrum, namely that (i) a “conservative” plant functional strategy is characterized by high initial investment in leaf construction, low leaf N, and low SLA values that produce low rates of carbon return for sustained periods whereas (ii) an “acquisitive” strategy is characterized by low initial investment, high leaf N, and high SLA values for high, but short-lived rates of return [2,5]. These traits in turn traded off with condensed tannins such that “acquisitive” oak species (high leaf N and SLA) were less chemically defended compared to “conservative” species which were more defended but had less leaf N and lower SLA. This result is consistent with the predictions of the Resource Availability Hypothesis which posits that foliar investment in anti-herbivore defenses should trade off with rapid leaf growth [9,41]. The fact that condensed tannins were the only phenolic compounds negatively associated with resource use traits might be explained by their high carbon content and molecular mass (e.g. relative to hydrolyzable tannins or flavonoids) which more quickly consume carbon resources that could otherwise be allocated towards leaf structural or functional traits [11].

Interestingly, *Q*. *agrifolia*, *Q*. *ilex* and *Q*. *suber* were the only evergreen species included and these tended to separate (especially the first two) from the others based on PC1 (see Fig 1). These species were located on the conservative side of the functional spectrum, consistent with previous reports indicating that evergreens exhibit a more conservative strategy of resource use relative to deciduous species [42,43]. This pattern illustrates that simultaneous expression and trade-offs in leaf traits can be summarized in different oak life histories reflecting contrasting patterns of adaptation to biotic and abiotic pressures. At the same time, however, we also observed broad variation in trait values for the deciduous species along PC1, from *Q*. *faginea* and *Q*. *pubescens* (marcescent species that retain dead foliage during winter) which are more similar to evergreens in their trait values to species such as *Q*. *robur* and *Q*. *rubra* which were fully on the acquisitive side of the Plant Economic Spectrum. This shows that deciduous species exhibit broad variation in functional strategies, perhaps more so than evergreen species, an observation that requires further testing.

Plant defense traits were distributed among multiple independent axes of trait variation in our ordination. The second axis of leaf traits was associated mainly with leaf lignin and flavonoid concentrations (and phosphorus), whereas the third axis of variation was largely driven hydrolysable tannins. Together with condensed tannins loading on the first axis, these results indicate that oaks may occupy a larger range of chemical defense trait space than resource acquistion trait space. Although the first two axes included resource acquistion traits, the third axis makes it clear that defense traits may vary in how strongly they are constrained by allocations to resource acquisition traits. It remains unknown whether a broader range of defensive traits such as trichomes and leafing phenology [18], as well as the differences between induced and constitutive levels [6] of all defenses may offer additional dimensions to plant trait space, or whether allocation contraints induce strong correlations between these traits and either resource acquisition or other defense traits. A fuller characterization of the dimensions of defensive investment is needed to appropriately test this idea.

Our results further emphasize the key role of climatic variables in shaping patterns of interspecific variation in oak leaf traits. Temperature was negatively associated with PC1, indicating that species found in warmer climates produce leaves with less N, lower SLA and more condensed tannins, whereas those found in colder climates exhibited oppostive values for these traits. Likewise, precipitation also exerted a significant influence on leaf trait variation based on its association with PC1 and PC2, such that oak species distributed in wetter climates exhibit a more acquisitive strategy and are less defended (high N, high SLA and low condensed tannins based on PC1, and low flavonids and lignins based on PC2), whereas those found in drier climates have a more conservative strategy and are better defended. These findings align with previously reported patterns of interspecific variation in defenses within *Quercus* [18], and further emphasize the role of climatic controls over co-variation in defensive and resource use traits. Results for PC2 also indicated a negative association between leaf P and precipitation which has been reported previously [44], and this could be explained by runoff or leaching of this nutrient from soils found in sites with higher precipitation. Finally, climate had no detectable influence on PC3 which was driven by variation in hydrolysable tannins, perhaps because these compounds play a less important role in plant biotic (or abiotic) resistance in oaks and are therefore less likely to vary in response to changing environmental conditions [20,27].

An important caveat to our results is that we observed these trait associationsin one-year old oak saplings and as such our analyses do not account for ontogenetic trait variation. Patterns of plant trait co-expression during juvenile stages of development are usually strongly linked with fitness and adaptation to specific environmental conditions [45]. However, functional strategies and growth-defense trade-offs may vary with plant ontogeny, and studies have shown that resource use strategies in adult trees may not always be concordant with those present during juvenile stages [46]. For example, McManus Chauvin et al. [11] reported that tree species with a “pioneer” lifestyle as juveniles appeared to mantain acquisitive strategies as adults, whereas other species with more conservative strategies as juveniles occupied different positions along the spectrum of resource use strategies as adults (see other examples in Kitajima et al. [47]). Future work comparing functional strategies across different ontogenetic stages is needed. In addition, a comparison of leaf patterns for other species-rich genera in Fagaceae (e.g. *Lithocarpus*) would yield broader insight into commonalities and differences in functional strategies in this taxonomic group.

In conclusion, plant defensive and resource use traits appear to be intrinsically—albeit not categorically—linked for the studied oak species, and we conjecture that this is likely to be the case for other plant taxa because of underlying trade-offs between growth and defense. Our findings also indicated that chemical variants within a single class (phenolic compounds) may separate into different axes of trait variation, suggesting multiple independent defensive strategies may emerge among species with similar chemistry. Further, we found that temperature and precipitation play key roles in determining interspecific patterns of relative allocation to different suites of traits. These findings call for further work evaluating the influence of biotic and abiotic factors, either through experimental or observational approaches, on all-inclusive multi-dimensional axes of interspecific variation in plant functional strategies.

## Supporting information

S1 Fig Oak species distribution ranges. Distribution ranges of each *Quercus* species. (a) *Quercus agrifolia*, (b) *Q*. *faginea*, (c) *Q*. *Ilex*, (d*) Q*. *macrocarpa*, (e) *Q*. *muhlenbergii*, (f) *Q*. *palustris*, (g) *Q*. *pubescens*, (h) *Q*. *pyrenaica*, (i) *Q*. *robur*, (j) *Q*. *rubra*, (k) *Q*. *suber*. Areas of distribution of each oak species are highlighted in red.
(PDF)

S2 Fig Quercus phylogenetic tree. Phylogenetic tree of the studied *Quercus* species based on Single Nucleotide Polymorphism matrices by ddRAD sequencing.
(PDF)
