# Supplementary figures and images for: Interspecific variation in leaf functional and defensive traits in oak species and its underlying climatic drivers
Source: PLoS One. 2018 Aug 20;13(8):e0202548. doi: 10.1371/journal.pone.0202548 (PMC6101385; doi:10.1371/journal.pone.0202548)

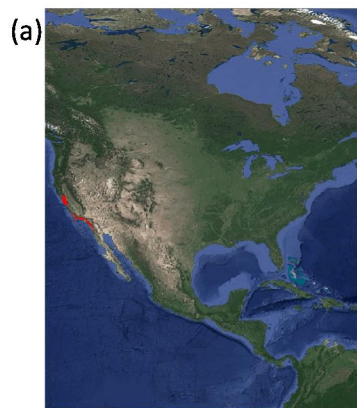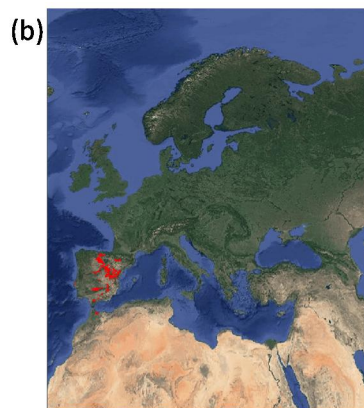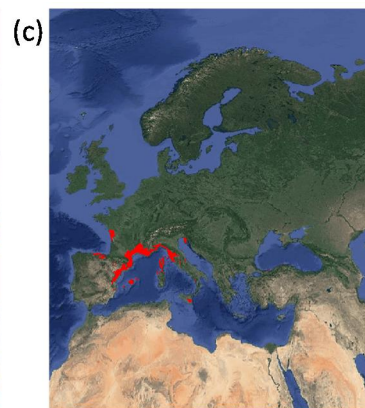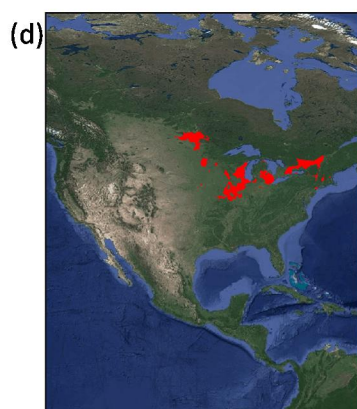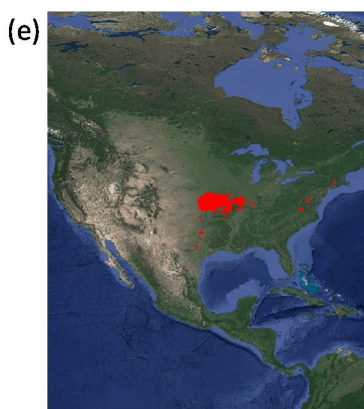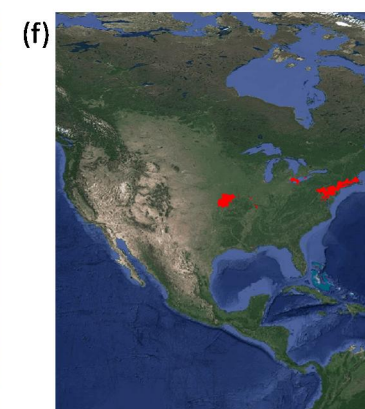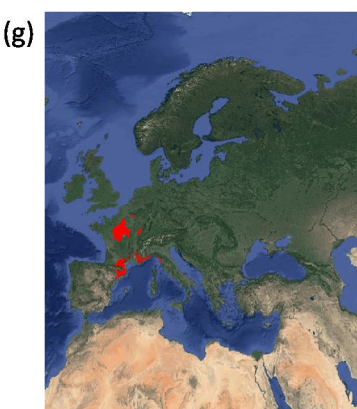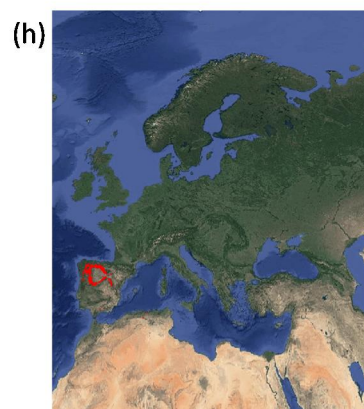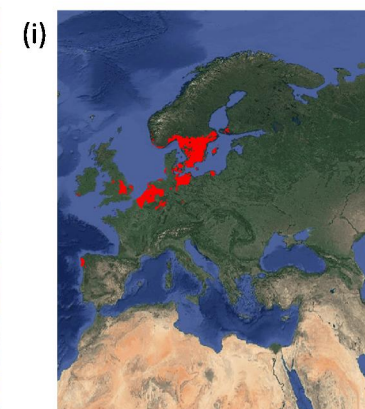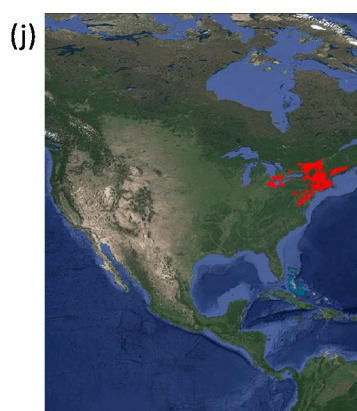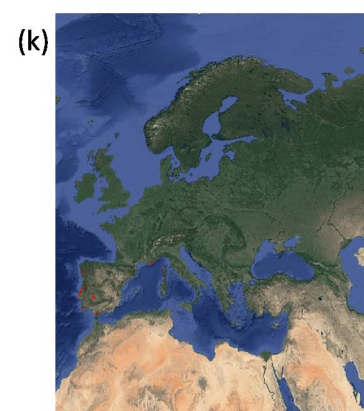

Supplement: S1 Fig — Distribution ranges of each Quercus species. (a) Quercus agrifolia, (b) Q. faginea, (c) Q. Ilex, (d) Q. macrocarpa, (e) Q. muhlenbergii, (f) Q. palustris, (g) Q. pubescens, (h) Q. pyrenaica, (i) Q. robur, (j) Q. rubra, (k) Q. suber. Areas of distribution of each oak species are highlighted in red. (PDF) [file pone.0202548.s001.pdf]

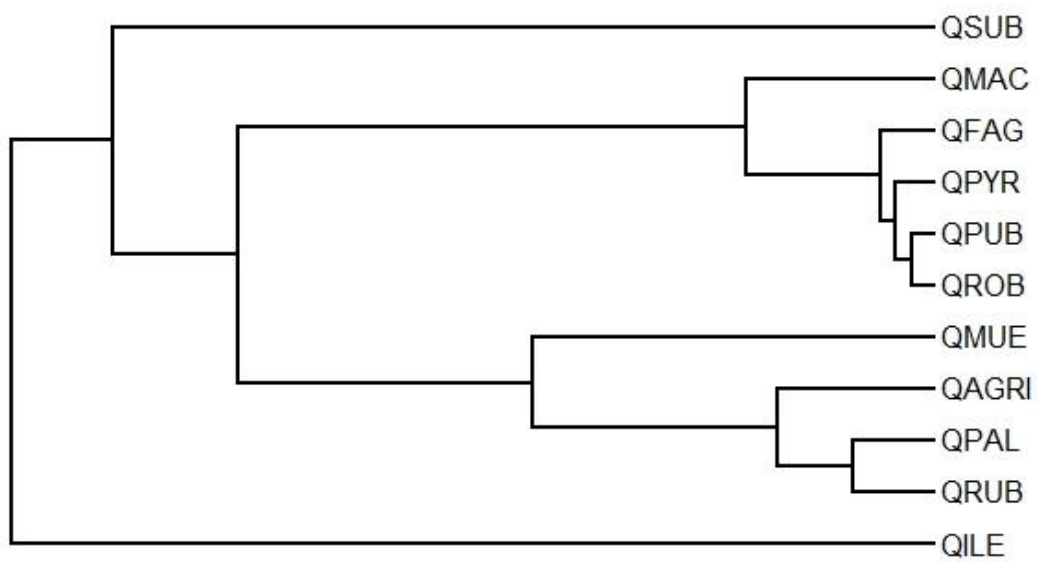

Supplement: S2 Fig — Phylogenetic tree of the studied Quercus species based on Single Nucleotide Polymorphism matrices by ddRAD sequencing. (PDF) [file pone.0202548.s002.pdf]
